# Occurrence and Genetic Parameters Estimation of Blood and Meat Spots in Brown-Shelled Eggs During the Extended Laying Period

**DOI:** 10.3390/ani16030404

**Published:** 2026-01-28

**Authors:** Honglei Jin, Bingxin Luo, Lin Xuan, Runzhe Wang, Jiahui Lai, Guiyun Xu, Jiangxia Zheng

**Affiliations:** National Engineering Laboratory for Animal Breeding and MOA Key Laboratory of Animal Genetics and Breeding, College of Animal Science and Technology, China Agricultural University, No. 2 Yuanmingyuan West Road, Haidian District, Beijing 100193, China; y202265855@163.com (H.J.); luobingxin0722@163.com (B.L.); linxuan@cau.edu.cn (L.X.); wangrunzhe@cau.edu.cn (R.W.); iamljhui@163.com (J.L.); ncppt@cau.edu.com (G.X.)

**Keywords:** egg, blood spot, meat spot, heritability, genetic correlation

## Abstract

Blood and meat spots are key internal egg quality indicators, and clarifying their genetic characteristics in late laying periods is critical for quality improvement via selective breeding. This study collected 392 eggs from 421 96-week-old Rhode Island Red hens across 69 families, analyzing 10 traits including blood/meat spots and standard egg quality traits. Heritability, genetic and phenotypic correlations were estimated using the DMU package. The incidences of blood and meat spots were 15.8% and 64.8%, respectively. Blood spots were yolk-localized, single and <1 mm in diameter, while meat spots mostly occurred on chalazae (83.5%) and thick albumen (33.1%), mostly multiple (56.1% with 2–5 spots) and 1.80 ± 1.53 mm in diameter (30% >2 mm). Blood spots had low heritability (0.05), meat spots moderate heritability (0.20). The two traits showed high positive genetic correlation (r_G_ = 0.93), and strong negative genetic correlations with albumen height and eggshell strength. In conclusion, blood and meat spots in late-laying hens differ in distribution, size and number, and meat spots are amenable to genetic selection for internal egg quality enhancement.

## Simple Summary

Blood and meat spots are visible inner defects in eggs that reduce consumer acceptance and economic value, particularly in aged laying hens producing brown-shelled eggs. With modern laying cycles extended to later ages, understanding why these defects occur and whether they can be reduced through breeding is important. In this study, a total of 392 eggs from Rhode Island Red hens were included in the analysis and examined for the presence, number, size, and location of blood and meat spots. Blood spots were few, very small, and mainly located on the yolk, while meat spots were common, often multiple, and mostly found in the egg white and chalaza. Genetic analysis showed a strong relationship between blood and meat spots, but their potential for improvement differs: meat spots had moderate heritability and could be reduced through selective breeding, whereas blood spots showed low heritability, suggesting that nutrition and management strategies may be more effective. These defects were linked to poorer internal egg quality, highlighting the importance of managing meat spots to improve egg quality, reduce economic losses, and support sustainable egg production.

## 1. Introduction

Internal egg quality is a key factor affecting the commercial value of eggs [1]. Among various internal defects, blood spots and meat spots are the most noticeable and undesirable for consumers. These defects typically appear as blood or tissue-like inclusions in the yolk or albumen. They reduce the visual quality and consumer acceptability of eggs and often lead to downgrading, resulting in the loss of the premium price associated with Grade A eggs [2]. Additionally, the occurrence of these defects may reflect abnormal physiological conditions in the reproductive system of the hen [3,4]. Studies have shown that brown-shelled eggs have a higher occurrence of blood and meat spots than white-shelled eggs, with reported occurrence rates of up to 18% [5,6]. In contrast, the frequency of these defects in white-shelled eggs has been reduced to approximately 0.5% through long-term phenotypic selection [2]. This reduction suggests that targeted genetic improvement could be an effective strategy for managing these traits in brown-shelled egg production.

The occurrence of blood and meat spots increases with the age of brown hens [7]. Wu et al. [6] reported that the frequency of blood and meat spots increased by approximately 20% between 22 and 67 weeks of age. Currently, the traditional 72–80-week laying cycle for egg-laying hens has been extended to 90–100 weeks globally [8]. To meet the dual demands of efficiency and sustainability in the industry, precise selection for blood and meat spot traits is essential. These defects are thought to be associated with localized hemorrhage or tissue sloughing in the ovary or oviduct [9]. Therefore, their occurrence and genetic characteristics warrant particular attention in poultry breeding programs.

Previous studies have indicated that the occurrence of blood and meat spots is influenced by genetic factors, not only between white- and brown-shelled egg lines but also within different strains of the same shell color type. For example, Campo and García Gil [2] selected hens from two different brown-shelled egg lines in Spain: Barred Red Vasca and Red Villafranquina. In both lines, the proportion of hens producing eggs with blood and meat spots was approximately 8%, significantly lower than the reported range for commercial brown-shelled egg strains (usually 16–21%). In contrast, Snyder [10] reported a much higher occurrence (59%) in Barred Plymouth Rock hens. These marked differences among genetic backgrounds suggest that the occurrence of blood and meat spots may be under genetic control, potentially involving major effect genes. However, despite evidence for genetic variation, most previous studies have treated blood spots and meat spots as a single, combined phenotype [2,6]. This approach implicitly assumes a shared biological and genetic basis, whereas blood spots and meat spots are generally considered distinct entities that differ in their origin, appearance, and temporal development during egg formation [4]. Consequently, combining these two traits may obscure trait-specific distribution patterns and genetic parameters. To date, studies that independently evaluate the genetic architecture of blood spots and meat spots in brown-shelled egg layers remain scarce, which may limit progress in effectively reducing the occurrence of these defects in shell eggs through genetic improvement.

Therefore, the aim of this study was to estimate the genetic parameters of blood spots and meat spots separately in brown-shelled eggs. Using large-scale phenotypic data, we systematically investigated their occurrence, distribution patterns, and relationships with major egg quality traits. This separate examination of blood spots and meat spots will provide a more accurate understanding of the genetic foundations and physiological mechanisms of these traits, offering scientific guidance for future precision breeding and production management.

## 2. Materials and Methods

### 2.1. Ethical Statement

All experimental procedures involving animals were approved by the Animal Care and Use Committee (AW80106202-1-03) of China Agricultural University, Beijing, China.

### 2.2. Animals and Data Collection

A total of 421 Rhode Island Red hens from the 20th generation of the breeding population were used. These hens were derived from 69 paternal half-sib families and 192 dams, resulting in a structured pedigree suitable for genetic parameter estimation. All hens were housed in individual cages with free access to feed and water and were managed under standardized feeding protocols. The temperature of the house was maintained at 24 ± 1 °C, and the hens were kept under a controlled environment with a lighting regimen of 16 h light and 8 h dark (16L:8D). At 96 weeks of age, one egg was collected from each hen. Broken and deformed eggs were excluded, and a total of 392 eggs were retained for phenotypic evaluation. Egg quality traits were measured within 24 h after egg collection.

Our heritability estimates were based on a single egg measurement per hen. Although this approach has limitations, from a quantitative genetic perspective, repeatability represents the proportion of phenotypic variance attributable to additive genetic and permanent environmental effects [11]. By definition, repeatability is greater than or equal to heritability. Previous studies have reported heritability estimates for blood and meat spot traits ranging from approximately 0.07 to 0.60 [12,13], suggesting that the occurrence of blood and meat spots likely exhibits low to moderate repeatability. This may provide some support for the reliability of using single measurements to estimate genetic parameters [14].

### 2.3. Traits Measurements

Blood spots and meat spots were assessed according to the criteria described by Wu et al. [6]. Under these criteria, even small red or brownish substances observed in the egg contents were classified as blood or meat spots. The location of blood and meat spots was recorded according to egg components, including albumen (outer thin albumen, inner thin albumen, and thick albumen), chalaza, and yolk. The frequency of blood and meat spot occurrence was subsequently calculated. The number and size of blood and meat spots in each egg were recorded. Spot size was defined as the maximum cross-sectional length of the spot. Egg quality traits measured included egg weight (EW), eggshell thickness (EST), eggshell strength (ESS), yolk weight (YW), albumen height (AH), Haugh unit (HU), yolk color (YC), and yolk ratio (YR). ESS was measured using the Model-II eggshell strength tester (Robotmation, Tokyo, Japan). EW, YW, AH, HU, and YC were measured using the EMT-5200 multi-function egg tester (Robotmation, Tokyo, Japan). EST was measured using a micrometer screw gauge at three positions (blunt end, equator, sharp end), and the average value was calculated. YR was calculated as the ratio of YW to EW.

### 2.4. Statistical Analysis

Descriptive statistics for blood and meat spot traits were performed using SPSS software (version 25.0, SPSS Inc., Chicago, IL, USA), with final data presented as mean ± standard deviation ($\bar{x}$ ± SD). For each egg quality trait at the same age, the arithmetic mean ± 3 SD was calculated, and values outside this range were removed as outliers. Trends in mean and quantile values of each trait across ages were described.

Phenotypic measurements from the Rhode Island Red population, together with pedigree information, were imported into the DMU software (v6-R5-2-EM64T, Aarhus University, Tjele, Denmark). Variance components for conventional egg quality traits were estimated using univariate animal models via restricted maximum likelihood (REML) implemented in the DMUAI module, which combines the average information (AI) algorithm with the expectation-maximization (EM) algorithm to ensure parameter estimates remain within the feasible parameter space. Heritability, genetic correlations, and phenotypic correlations were obtained, with all effects evaluated simultaneously. The univariate animal model used for conventional traits was:

y=Xb+Zu+e

where y is the vector of phenotypic observations, b is the fixed effects vector, and u∼N (0, ${A\sigma }_{u}^{2}$) represents the random effects vector, where e∼N (0, ${I\sigma }_{e}^{2}$) is the residual error, and I is the identity matrix. X and Z represent the incidence matrices for b and u, respectively.

Although the number and size of spots were recorded, these measurements showed highly skewed distributions. Consequently, the current data structure was not suitable for reliable variance component estimation. The primary objective of this study was to assess the genetic susceptibility to these defects rather than their severity. Accordingly, blood spot (BS) and meat spot (MS) were treated as binary traits (0 = absence; 1 = presence). Binary traits were analyzed using a threshold animal model, consistent with previous genetic studies on binary egg traits, such as poultry survival [15]. This approach provides robust and interpretable estimates of additive genetic variance by assuming that the observed binary phenotypes reflect an underlying continuous liability. The probability of observing a positive record was modeled using a probit link function, with the underlying liability modeled as:

$$\lambda_{ij}=\mu +{BATCH}_{j}+a_{k}+e_{ij}$$

where $\lambda_{ij}$ is the unobserved liability for the presence of blood or meat spots, μ is the overall mean, ${AGE}_{i}$ is the fixed effect of age class, ${BATCH}_{j}$ is the fixed effect of batch (*j* = 1, 2), $a_{k}$ is the random additive genetic effect of animal *k*, and $e_{ij}$ is the residual effect. Additive genetic effects were assumed to be normally distributed as a∼N (0, ${A\sigma }_{a}^{2}$), where A is the numerator relationship matrix, and residuals were assumed to follow a normal distribution as e∼N (0, ${I\sigma }_{e}^{2}$) with residual variance fixed to 1. Variance components and heritability on the underlying liability scale were estimated using the Bayesian threshold model implemented in DMU.

Data visualization was performed using “ChiPlot” (https://www.chiplot.online/).

## 3. Results and Discussion

### 3.1. Occurrence, Number, Size, and Distribution Characteristics of Blood Spots and Meat Spots

In this study, the occurrence of blood spots was approximately 15.8%, and the occurrence of meat spots was about 64.8% in 96-week-old Rhode Island Red hens. Blood spots were always single and located on the yolk, with a diameter of less than 1 mm. This finding is consistent with the observations of van Wagenen et al. [16], who also studied brown-shelled breeds and found that, except for a few large blood clots that spread into the albumen, almost 100% of blood spots were located on the yolk. However, Burmester and Card [17] reported that multiple blood spots (2–3 spots per egg) were observed in 25–30% of eggs in some hen strains. The differences in findings might be due to variations in specific populations.

**Figure 1:**
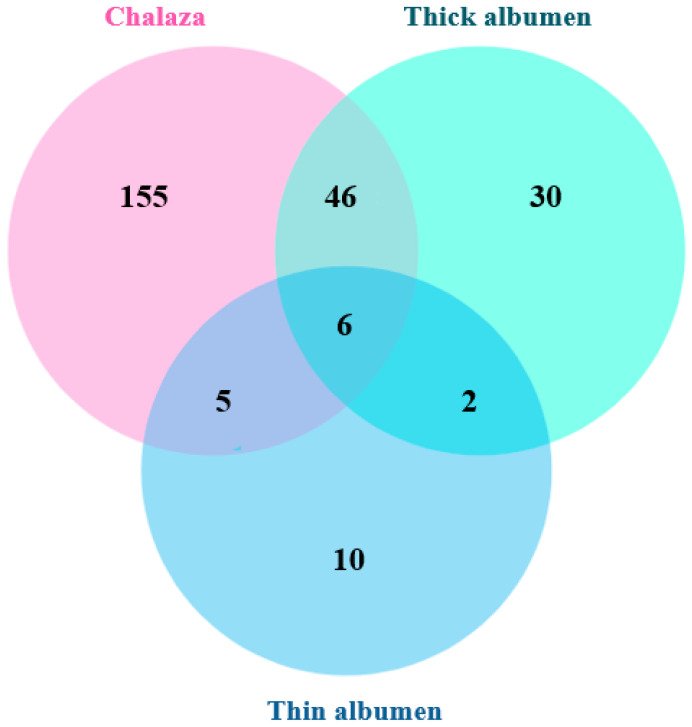
Meat spots occurring individually or in combination in the chalaza, thick albumen, and inner thin albumen.

The distribution characteristics of meat spots is shown in Figure 1. Most meat spots were located on the chalaza and thick albumen, with a few found in the thin albumen. Specifically, 83.5% of the meat spots were located in the chalaza, 33.1% in the thick albumen, and 9.1% in the thin albumen. Additionally, 20.5% of the meat spots occurred in both the thick albumen and chalaza, with a very small number present in all three locations. This finding is consistent with previous studies [16], which reported that nearly 9 out of 10 meat spots were located in the albumen or wrapped around the chalaza. In the meat spot eggs identified in this study, 43.9% contained only one meat spot, 13.6% contained two meat spots, 4.5% contained three to four spots, and 37.9% contained five or more meat spots.

**Figure 2:**
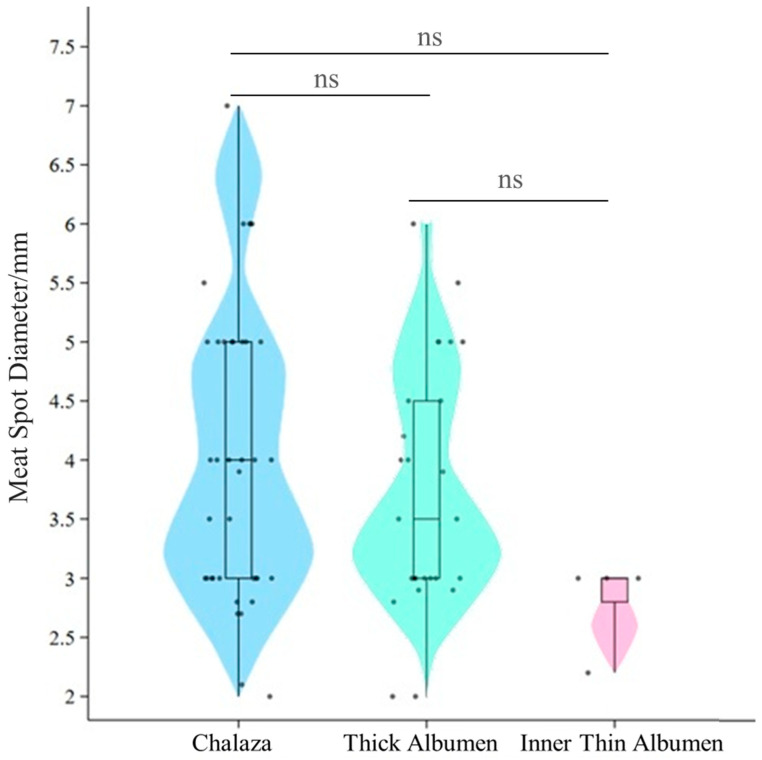
Distribution of meat spot diameters (≥2 mm). *ns* indicates no statistically significant difference (*p* > 0.05).

This study found that, within the visible range, the average diameter of meat spots was 1.80 ± 1.53 mm. Among them, meat spots with a diameter between 1 and 2 mm accounted for about 70%. According to the USDA and GB/T 39438-2020 standards [18], meat spots larger than 2 mm should be removed. Further statistical analysis of meat spots with a diameter day ≥ 2 mm is shown in Figure 2. These larger meat spots accounted for approximately 30%, with an overall average diameter of 3.84 ± 0.14 mm. Specifically, the average diameters were 4.04 ± 0.20 mm in the chalaza, 3.71 ± 0.20 mm in the thick albumen, and 2.80 ± 0.20 mm in the thin albumen. No significant differences were found in the diameters among these three locations. Meat spots with diameters in the range of [2, 5) mm appeared with an approximately equal probability in the chalaza and thick albumen, whereas meat spots with diameters ≥ 5 mm were almost exclusively found in the chalaza.

Larger meat spots were observed to be less frequent. They tended to be concentrated in specific areas, such as the chalaza. In contrast, smaller spots were more evenly distributed between the chalaza and the thick albumen. This distribution pattern suggests that smaller and larger spots may form through distinct mechanisms. The negative correlation between the number and size of meat spots further supports the hypothesis that smaller spots may form more frequently but remain smaller, while larger spots, which are less frequent, grow in specific areas with higher severity.

Blood spots are mainly distributed on the yolk, which may reflect ovarian hemorrhage. The infundibulum may also be an important site for blood spot occurrence. Similarly, Meat spots may be associated with oviductal damage at the infundibulum. Wu et al. [6] conducted histological examinations of blood and meat spots and observed red blood cells, inflammatory cells, and necrotic cells. These findings suggest that the defects may be associated with ruptures of blood vessels in the ovary or oviduct during ovulation. Additional histological evidence indicates that blood and meat spots may be associated with inflammatory responses in the ovary and the magnum of the oviduct.

Our study found that meat spots were primarily located in the chalaza and thick albumen, with larger meat spots (≥5 mm in diameter) mostly found in the chalaza. The infundibulum and magnum are responsible for the formation of the chalaza and thick albumen, respectively. Thus, damage to the infundibulum may be a major cause of meat spot occurrence. However, previous studies have also explored this issue [9,17,19].

Honkatukia et al. [20] found through histopathological analysis that blood spots are mainly composed of red blood cells, while meat spots consist of necrotic tissue or epithelial cell debris from the reproductive tract. These two types of spots originate from different locations and mechanisms. Early research also suggested that some meat spots may originate from degraded blood spots, but their occurrence processes and mechanisms are distinct [21]. Furthermore, blood spots are commonly found on the yolk surface, while meat spots are often located in the albumen. The occurrence of these spots varies significantly under different shell colors, hen ages, and nutritional conditions [6,20].

The distinct histological origins, occurrence mechanisms, and physiological implications of blood and meat spots suggest that they should be measured and analyzed separately in research. This study provides valuable insights into the entire egg-laying process and the health of the reproductive tract in hens, helping to identify factors that contribute to tissue damage and offering new approaches to improving egg quality and reducing the occurrence of meat spots.

### 3.2. Heritabilities, Genetic and Phenotypic Correlations

**Figure 3:**
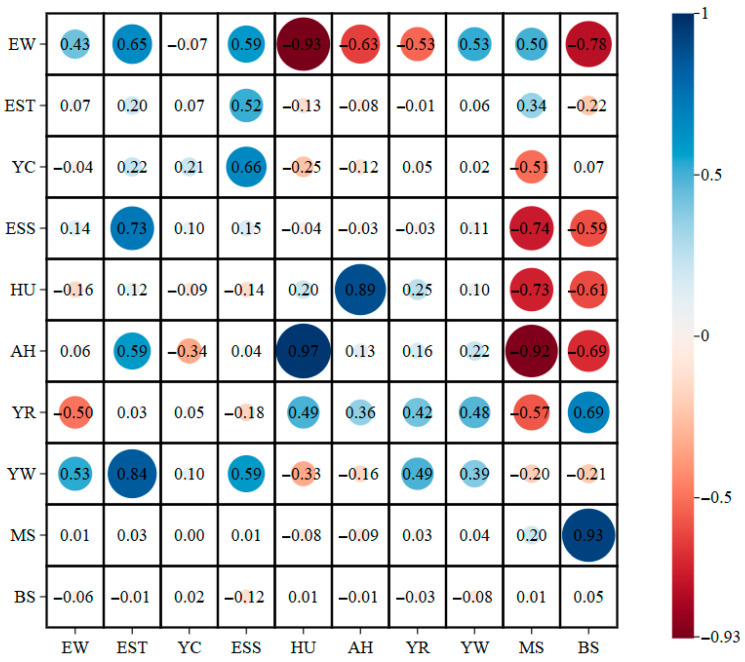
Genetic and phenotypic correlations between egg quality traits of Rhode Island Red. Abbreviations: EW, egg weight; EST, eggshell thickness; YC, yolk color; ESS, eggshell strength; HU, haugh unit; AH, albumen height; YR, yolk percentage; YW, yolk weight; MS, meat spots; BS, blood spots. The upper triangle represents genetic correlation, while the lower triangle represents phenotypic correlation, and the diagonal line shows the heritability of each trait.

The results of the genetic parameter estimation are shown in Figure 3. In this study, the heritability of blood spots was estimated to be 0.05, which is consistent with previous studies. Honkatukia et al. [20] conducted a genetic evaluation of blood spots in eggs using genomic tools and reported a heritability of approximately 0.05. Similarly, Lerner et al. [22] pointed out that the heritability of blood spots varies among different strains but generally falls between 0.15 and 0.40. Although the heritability is low, it still suggests that blood spots have a genetic basis. In contrast to our results, Becker and Bearse [13] reported a slightly higher heritability of 0.16 for blood spots, and when vitamin A was deficient, the heritability increased to 0.60.

Blood and meat spots in eggs can lead to variations in the microbial community [6], increasing the risk of infection by pathogens such as Salmonella and reducing hatching rates [23,24]. However, there are fewer studies on the genetic parameters of meat spots. The heritability of meat spots in this study was estimated at 0.20, indicating a moderate genetic level. Honkatukia et al. [20] conducted variance analysis on the combination traits of meat spot quantity and size in Lohmann Brown hens, with a heritability estimate of only 0.01, indicating that this trait is mainly influenced by environmental and individual physiological factors, with little additive genetic effect. However, Becker and Bearse [13] reported that the heritability of internal contents such as meat spots ranged from 0.07 to 0.60, suggesting large differences across populations, evaluation standards, and variance component estimation methods. This result aligns with our study and indicates that meat spots are a relatively heritable egg abnormality. In breeding programs, the heritability of meat spots can serve as an important reference for optimizing egg quality, particularly in breeding plans aimed at improving egg quality and economic efficiency.

Genetic and phenotypic correlations between blood and meat spots and other egg quality traits are presented in Figure 3. The results show that the genetic correlations between blood and meat spots and the measured egg quality traits ranged from −0.982 to 0.929. There is a high positive genetic correlation between BS and MS (r_G_ = 0.93), which is consistent with previous studies. In the study by Honkatukia et al. [20], the genetic correlation between the blood spot and meat spot combinations was 0.83. This suggests that these two traits are genetically regulated by shared or tightly linked genes.

BS and MS showed strong negative genetic correlations with AH and HU (r_G_ = −0.69, r_G_ = −0.61; r_G_ = −0.92, r_G_ = −0.73), respectively. This indicates that the occurrence of blood and meat spots is positively correlated with age. As the hen’s age increases, its egg-laying function gradually declines, and the albumen becomes thinner. Both AH and HU tend to decrease with age [25,26,27]. Previous studies indicate that age-related changes in the oviduct, such as the accumulation of reactive oxygen species (ROS), may contribute to epithelial cell damage. Oxidative stress may further impair oviduct function and potentially reduce thick albumen secretion [28,29,30]. Oviductal damage may also be associated with the shedding of tissue fragments, which could contribute to meat spot occurrence. Therefore, balancing the relationships between these traits is crucial in breeding programs.

There were significant negative correlations between MS, BS, and ESS (r_G_ = −0.74, r_G_ = −0.59), indicating that the increase in blood and meat spots is related to a decline in eggshell quality. The eggshell is formed in the uterine part of the oviduct, and the uterine proteins or metabolic products can affect the calcification process of the eggshell [31,32], which ultimately determines the eggshell quality [33,34]. The health of the oviduct is closely linked to the eggshell quality [30]. Therefore, this study hypothesizes that the occurrence of blood and meat spots reflects functional abnormalities in the hen’s oviduct, which interfere with the normal deposition of minerals in the shell gland, resulting in uneven calcium salt distribution and disordered crystal arrangement, ultimately affecting the mechanical integrity of the eggshell.

This study found that blood spots are primarily distributed on the yolk, but their overall occurrence is low, and their heritability is also low. This suggests that genetic improvement through selective breeding alone would be inefficient, and nutritional regulation could be a more feasible intervention. Previous studies have shown that vitamin K is involved in regulating blood coagulation to reduce the risk of capillary hemorrhage during ovulation, and selenium enhances antioxidant capacity to reduce oviduct tissue damage. Therefore, the addition of vitamin A, vitamin K, and trace element selenium to feed could be considered [1,35]. Meat spots, on the other hand, are primarily distributed in the yolk chalaza and thick albumen. Meat spots with a diameter ≥ 2 mm are relatively more common. The occurrence of meat spots is associated both with local tissue damage caused by oviduct inflammation and with the physiological decline caused by oviduct aging. The heritability of meat spots is 0.20, indicating moderate heritability and potential for genetic improvement.

Current research focuses on non-destructive detection technologies. Blood spots can be detected using spectral technology that identifies hemoglobin characteristic signals. However, due to interference from the eggshell pigment (protoporphyrin) in brown eggs, this technology is not applicable. Therefore, effective non-destructive detection methods for meat spots are still lacking [36,37]. A breakthrough in non-destructive detection technology for meat spots will provide key technical support for directed selection through phenotypic culling and significantly accelerate the breeding of low meat spot chicken lines.

## 4. Conclusions

In this study, brown-shelled laying hens with extended laying periods showed a high occurrence of blood and meat spots. The frequencies reached 15.8% for blood spots and 64.8% for meat spots. Significant differences were observed in the location, size, and number of these spots. These findings indicate that blood spots and meat spots may need to be measured separately in genetic analyses. The heritabilities of blood and meat spots were estimated at 0.05 and 0.20, respectively. A high genetic correlation was observed between the two traits (r_G_ = 0.93). These results suggest that meat spots may be amenable to genetic improvement. For blood spots, nutritional and physiological interventions may be more effective, although this requires further investigation. Analyzing the genetic correlations between these traits and other egg quality traits may provide valuable insights for breeding strategies. Meat spots were primarily located in the chalaza and thick albumen. This distribution may reflect tissue shedding in the infundibulum and magnum of the oviduct, which could contribute to meat spot occurrence. Overall, these findings may help inform genetic breeding programs aimed at improving the quality of brown-shelled eggs. The entire concluding paragraph contains dense, information-heavy sentences influenced by non-native syntax. Shorter sentences and a clearer separation between data-supported findings and broader implications are recommended.

## Data Availability

The relevant data of this study are included in the article. If you have any further questions, please contact the first author of the article.
